# Global Trends in the Epidemiology and Management of Dyslipidemia

**DOI:** 10.3390/jcm11216377

**Published:** 2022-10-28

**Authors:** Tianxiao Liu, Dong Zhao, Yue Qi

**Affiliations:** Center for Clinical and Epidemiological Research, Beijing Anzhen Hospital, Capital Medical University, Beijing Institute of Heart, Lung and Blood Vessel Diseases, The Key Laboratory of Remodeling-Related Cardiovascular Diseases, Ministry of Education, Beijing Municipal Key Laboratory of Clinical Epidemiology, Beijing 100029, China

**Keywords:** dyslipidemia, epidemiology, guidelines, therapy, management

## Abstract

Dyslipidemia, especially a circulating non-optimal level of cholesterol, is one of the most important risk factors for atherosclerotic cardiovascular disease (ASCVD), which accounts for the most deaths worldwide. Maintaining a healthy level of blood cholesterol is an important prevention strategy for ASCVD, through lifestyle intervention or cholesterol-lowering therapy. Over the past three decades, the epidemiology and management of dyslipidemia has changed greatly in many countries. Therefore, it is necessary to understand the current epidemiologic features of dyslipidemia and challenges from a global perspective.

## 1. Introduction

Circulating non-optimal cholesterol, including increased low-density lipoprotein cholesterol (LDL-C) and remnant cholesterol carried by triglyceride-rich lipoproteins, comprises the main type of dyslipidemia worldwide and is a major risk factor for atherosclerotic cardiovascular disease (ASCVD) [1]. It has been well demonstrated that atherosclerosis is causally associated with the retention of low-density lipoprotein (LDL) in the intima, which is the main deliverer of cholesterol to the focal areas of the artery wall. Cholesterol in LDL induces the activation of vascular endothelial cells, which subsequently recruit monocytes into the sub-endothelial space, and promote macrophage activation and inflammatory response in the arterial intima. Furthermore, these macrophages engulf LDL in the intima and turn into foam cells, giving rise to atherosclerotic lesions and eventually triggering ischemic heart disease (IHD), ischemic stroke, and other ASCVDs [2,3,4]. Additionally, remnant cholesterol carried by triglyceride-rich lipoproteins is also recognized to play a causal role in atherosclerosis development, similarly to LDL-C [5]. Non-high-density lipoprotein cholesterol (non-HDL-C), including LDL-C and remnant cholesterol, has been considered as cholesterol carried by both atherogenic lipoproteins. Therefore, LDL-C and non-HDL-C have been widely used as lipid-lowering targets. Effective treatment strategies for lowering LDL-C, including statin monotherapy, statin with ezetimibe, or statin with proprotein convertase subtilisin/kexin type 9 (PCSK9) inhibitors, have contributed to the reduction of further ASCVD events proportional to the absolute reduction in LDL-C [6,7,8]. However, ASCVD still remains the top cause of death globally, despite advances in controlling dyslipidemia in many countries. It is important to understand the global epidemiologic features and advances in the management of dyslipidemia, so as to identify the key issues or barriers of alleviating current and future burdens of dyslipidemia-related disease. Many studies have reported on the epidemiology trends in dyslipidemia and its management at the country or region level [9,10], and a few studies have analyzed lipid levels and changes overtime on a global scale [11,12]. However, a review article summarizing the current knowledge combined with the epidemiology and management of dyslipidemia from a global perspective is lacking. Therefore, in this review, we will summarize the developments in global trends in the epidemiology and management of dyslipidemia.

The data presented in this review are mainly identified from four sources. The first data source is an open database of population-based data on risk factors collected by the Non-communicable diseases (NCD) Risk Factor Collaboration (NCD-RisC) [13], a worldwide network of health scientists that provides rigorous and timely data. The second data source is the open database of the Global Burden of Disease (GBD) study in the Global Health Data Exchange of the Institute for Health Metrics and Evaluation [14], with available information on the mortality rate, number, and proportion of deaths from various diseases at global, regional, and country levels. The third source is the MEDLINE database, which we searched for relevant publications regarding the epidemiology and management of dyslipidemia in the past 10 years, studies on the burden of disease associated with dyslipidemia, and country-specific guidelines on dyslipidemia management. The final source is the international guideline library of the Guidelines International Network (GIN) [15], which contains the latest guidelines across the globe.

Through investigation of the available data and published reports, we identify features of the global trends on the epidemiology and management of dyslipidemia worldwide, as described below.

## 2. Global Epidemiology of Dyslipidemia

The NCD-RisC study is the largest worldwide study so far on the global distribution of blood cholesterol and changing trends [13]. This study pooled data of 102.6 million individuals aged 18 years and older collected from 1127 population-based studies in 200 countries and territories, including 48 studies from Sub-Saharan Africa; 28 from Central Asia, the Middle East, and North Africa; 6 from South Asia; 16 from East and Southeast Asia; 17 from Oceania; 3 from high-income Asia-Pacific countries; 35 from Latin America and the Caribbean; 27 from high-income Western countries; and 20 from Central and Eastern Europe. The NCD-RisC study provided comparisons of the age-standardized mean total cholesterol (TC) and non-HDL-C among countries and regions from 1980 to 2018 [13]. Globally, little or no change was observed between 1980 and 2018 in the global age-standardized mean of TC and non-HDL-C, yet substantial differences exist among countries and regions, as reported in the latest two articles [11,12].

**Figure 1:**
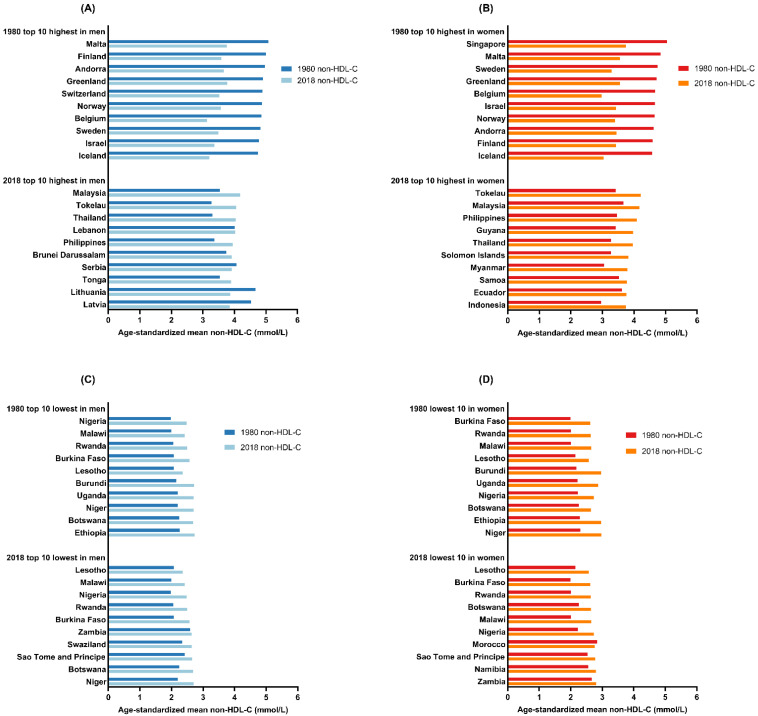
Top 10 countries with the highest and lowest age-standardized mean non-HDL-C levels in 1980 and 2018 for men and women. (**A**), Top 10 countries with the highest age-standardized mean non-HDL-C in men. (**B**), Top 10 countries with the highest age-standardized mean non-HDL-C in women. (**C**), Top 10 countries with the lowest age-standardized mean non-HDL-C in men. (**D**), Top 10 countries with the lowest age-standardized mean non-HDL-C for women. Data obtained from NCD-RisC study. Available online: https://www.ncdrisc.org (accessed on 1 July 2022) [13]. Abbreviations: non-HDL-C, non-high-density lipoprotein cholesterol.

Based on the data from the NCD-RisC study, we summarized non-HDL-C levels and their changes across countries and exhibited the features for the global epidemiology of non-HDL-C levels. Over the past four decades, the median value of the global age-standardized mean non-HDL-C was almost unchanged in men rising from 3.36 mmol/L [interquartile range, 2.82–3.90 mmol/L] in 1980 to 3.37 mmol/L [3.04–3.59] in 2018, and decreased slightly in women from 3.44 mmol/L [interquartile range, 2.83–3.91 mmol/L] in 1980 to 3.34 mmol/L [3.08–3.54] in 2018 across the 200 countries. However, the majority of countries with the highest levels of age-standardized mean non-HDL-C in 1980 experienced significant declines. As shown in Figure 1A,B, the top 10 countries with the highest age-standardized mean levels of non–HDL-C levels in 1980 were mainly high-income countries in Western Europe and Singapore, which had age-standardized mean levels of non–HDL-C of >4.7 mmol/L in men and >4.5 mmol/L in women.

**Figure 2:**
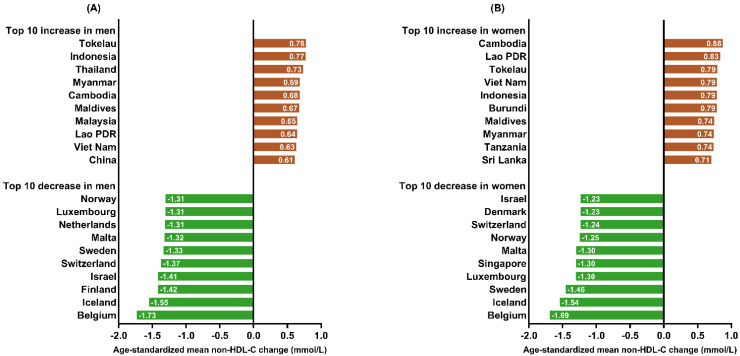
Top 10 countries with the largest increases and decreases of age-standardized mean non-HDL-C levels from 1980 to 2018 for men and women. (**A**), Top 10 countries with the largest increase and decrease of age-standardized mean non-HDL-C in men. (**B**), Top 10 countries with the largest increase and decrease of age-standardized mean non-HDL-C in women. Data obtained from NCD-RisC study. Available online: https://www.ncdrisc.org (accessed on 1 July 2022) [13]. Abbreviations: non-HDL-C, non-high-density lipoprotein cholesterol.

These countries underwent the largest reduction over the past four decades, with age-standardized mean levels of non-HDL-C decreasing more than 1.0 mmol/L from 1980 to 2018 (Figure 2).

The age-standardized mean levels of non-HDL-C in these European countries decreased to the global average level in 2018 (Figure 1A,B). By contrast, the age-standardized mean non–HDL-C in Singaporean women ranked the highest at 5.0 mmol/L in 1980 across 200 countries (Figure 1B), yet this number reduced to 3.7 mmol/L, which decreased to 11th, in 2018. On the other hand, the top 10 countries with the highest age-standardized mean non–HDL-C levels in 2018 were predominantly composed of developing countries from Southeast Asia, Western Asia, and Oceania. These countries had lower age-standardized mean non-HDL-C levels (<3.8 mmol/L) in 1980, and have experienced a large increase over the past four decades (Figure 2). In addition to these countries, others have also experienced a substantial increase. In particular, age-standardized mean non-HDL-C in Chinese men increased 0.61 mmol/L over 40 years, ranking from 153rd in 1980 to 99th in 2018, which reached or surpassed the non-HDL-C levels of some Western countries. As for the top 10 countries with the lowest age-standardized mean non-HDL-C levels in 1980, many countries in Africa registered no such changes and are still below the global average level over the past four decades (Figure 1C,D). These changes in age-standardized mean non-HDL-C diminished the variation of non-HDL-C among countries from 1980 to 2018. The upper quartile of global age-standardized mean non-HDL-C from 1980 to 2018 decreased, for men from 3.90 mmol/L to 3.59 mmol/L, and for women from 3.91 mmol/L to 3.54 mmol/L, while the lower quartile of age-standardized mean non-HDL-C from 1980 to 2018 increased, for men from 2.82 mmol/L to 3.04 mmol/L, and for women from 2.83 mmol/L to 3.08 mmol/L. Additionally, the standard deviation of global age-standardized mean non-HDL-C among the 200 countries from 1980 to 2018 decreased from 0.76 mmol/L to 0.38 mmol/L in men and from 0.69 mmol/L to 0.31 mmol/L in women. Genetic and endemic factors also have a great influence on blood cholesterol level [16]. However, the data of the NCD-RisC study is collected at the national level, which cannot describe the epidemiology of dyslipidemia in ethnically and culturally heterogeneous populations. It is necessary to explore the influence of genetic and endemic factors on the epidemiology of dyslipidemia in the future.

## 3. ASCVD Attributed to Dyslipidemia

**Figure 3:**
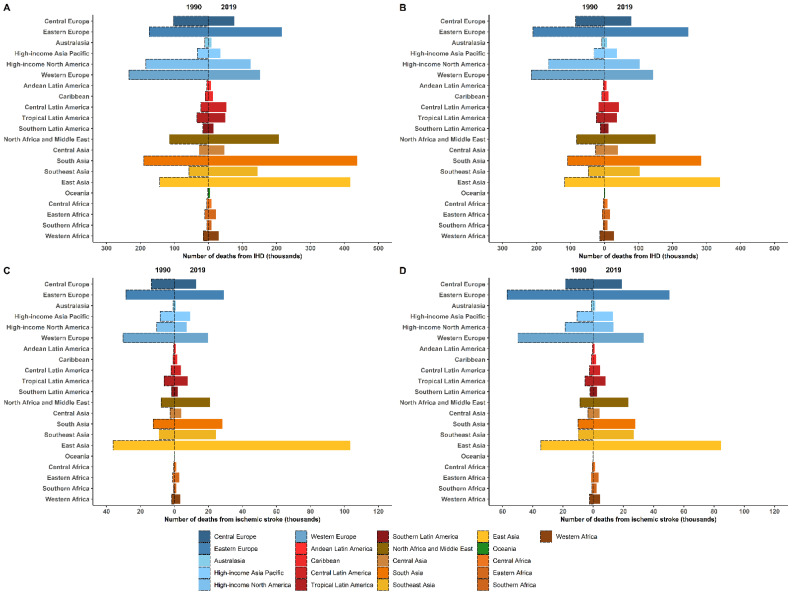
Change in deaths from ischemic heart disease and ischemic stroke attributable to high low-density lipoprotein cholesterol by region in 1990 and 2019. (**A**), Change in deaths from ischemic heart disease attributable to high low-density lipoprotein in men. (**B**), Change in deaths from ischemic heart disease attributable to high low-density lipoprotein in women. (**C**), Change in deaths from ischemic stroke attributable to high low-density lipoprotein in men. (**D**), Change in deaths from ischemic stroke attributable to high low-density lipoprotein in women. Data obtained from GBD database available on https://vizhub.healthdata.org/gbd-results/ (accessed on 1 July 2022) [14]. Abbreviations: IHD, ischemic heart disease.

The estimations of burden of ASCVD attributed to high LDL-C levels worldwide were based on data available in the GBD database [14]. According to the estimations of the GBD study in 2019, a total of 3.78 million deaths from IHD worldwide were attributable to high LDL-C levels, accounting for 44.3% of IHD deaths. While 0.61 million deaths from ischemic stroke were attributable to high LDL-C levels, accounting for 22.4% of ischemic stroke deaths [14]. Globally, these numbers have increased since 1990 (Figure 3).

The most important feature of this trend was the substantial increase in deaths attributable to high LDL-C levels in Asian countries. A consistent result was found in the NCD-RisC study [11]. The study reported that from 1990 to 2017, the number of deaths attributable to high non-HDL-C more than tripled in East Asia and more than doubled in Southeast Asia. The number of deaths attributable to high non-HDL-C in East, Southeast and South Asia accounted for 25% of deaths attributable to high non-HDL-C worldwide in 1990 and rose to about 50% in 2017.

**Figure 4:**
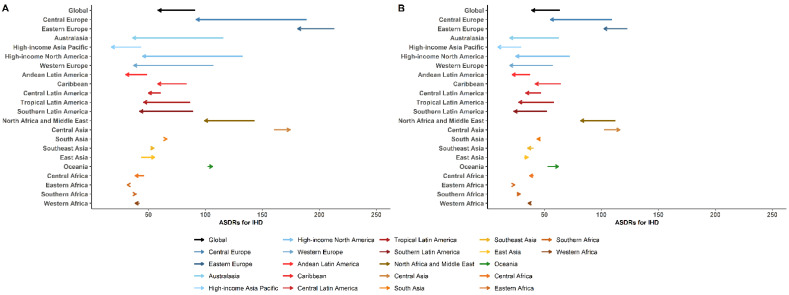
Change in age-standardized death rates per 100,000 of the general population from ischemic heart disease attributable to high low-density lipoprotein cholesterol between 1990 and 2019 by region for men (**A**) and women (**B**). Data obtained from the GBD database available on https://vizhub.healthdata.org/gbd-results/ (accessed on 1 July 2022) [14]. Abbreviations: ASDRs, age-standardized death rates; IHD, ischemic heart disease.

**Figure 5:**
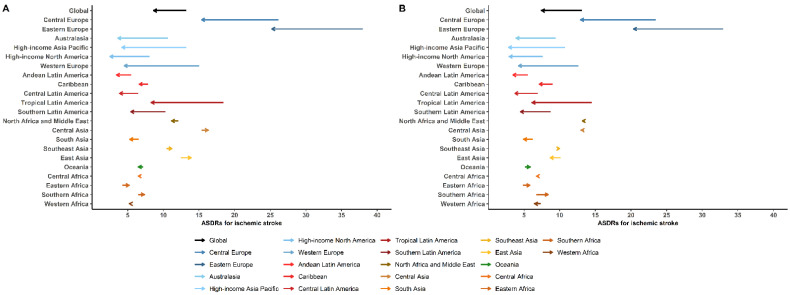
Change in age-standardized death rates per 100,000 of the general population from ischemic stroke attributable to high low-density lipoprotein cholesterol between 1990 and 2019 by region for men (**A**) and women (**B**). Data obtained from GBD database available on https://vizhub.healthdata.org/gbd-results/ (accessed on 1 July 2022) [14]. Abbreviations: ASDRs, age-standardized death rates.

Global age-standardized death rates (ASDRs) for IHD and ischemic stroke attributable to high LDL-C levels had a 35.1% reduction and 34.2% reduction in men, and a 38.1% reduction and 42.8% reduction in women over the last 30 years, respectively. Most Western countries/territories corresponded to the global trends, with a decrease in ASDRs attributable to high LDL-C over the past three decades. However, for Asian countries, the ASDRs for IHD and ischemic stroke attributable to high LDL-C did not decrease over the same 30 years and even significantly increased in Central Asia and East Asia (Figure 4 and Figure 5).

On the other hand, many African countries have experienced no change in ASDRs attributable to high LDL-C since 1990, and remained low in 2019. Considerable heterogeneity exists both within and between populations worldwide, despite the similarly huge and growing burden of cardiovascular disease (CVD), reaffirming the need to reprioritize the global management and control of non-optimal cholesterol within populations.

## 4. Management of Dyslipidemia

From a global perspective, the use of lipid-lowering medications in high risk people has been recommended by all relevant CVD prevention guidelines issued by professional societies of different countries or regions. The increasing availability and utility of statins have greatly contributed to the reduction of LDL-C related ASCVD burden. Education of the public to improve awareness about dyslipidemia-related CVD risk, increasing the opportunity for lipid testing and ASCVD risk assessment, and developing and updating clinical guidelines on dyslipidemia are also key issues to improve dyslipidemia management. In the past 20 years, such factors have resulted in considerable changes in lipid levels in many countries worldwide [17,18]. However, regions with different social demographic features show discrepancies in the management strategies of dyslipidemia.

### 4.1. Guidelines for Dyslipidemia Management

As highlighted in the data on the global epidemiology of dyslipidemia and burden of cardiovascular disease, there is considerable variation in dyslipidemia-related ASCVD risk across countries. Thus, the anticipated benefits in terms of CVD prevention as a consequence of effective implementation of either population-wide lifestyle change strategies or treatment of high risk individuals with cholesterol-lowering medication differ across ethnically and culturally heterogeneous populations. In this review, we summarize the differences in guidelines for the management of plasma lipid disorders issued by different countries or professional organizations [19,20,21,22,23,24,25,26,27,28,29,30,31,32,33,34,35]. Newly updated guidelines for most countries have been published after 2015 and are therefore based on relatively recent research evidence to provide time-sensitive guidance. Clinical guidelines have an essential role in guiding clinical practice by providing physicians with recommendation based on these latest data. As a guide to management strategies, there are some similarities in the guidelines issued by Western and non-Western countries. 

All guidelines support lifestyle modification as an effective method to manage lipid level. Most clinical guidelines across countries recommend treatment strategies as a function of CVD risk assessment and untreated LDL-C levels for the purpose of keeping LDL-C within the specific target values. Almost all guidelines recommend LDL-C as the primary treatment target, and non-HDL-C and/or apolipoprotein B as the secondary treatment target [19,20,21,23,24,27,30,34]. Additionally, statins are the first-line agents in all guidelines. The addition of ezetimibe is recommended by most countries when the LDL-C goal is not achieved with the maximum tolerated dose of statins. Due to the successes of recent clinical trials, PCSK9 inhibitors are also recommended in some guidelines [19,21,24,27,28,29,30,34]. Besides the two nonstatin widely recommended in some guidelines, bile acid sequestrants may be considered as an add-on drug with statins to reduce LDL-C. Fibrates are recommended to lower triglyceride levels [21,23,24,25,27,28,29,30,34], but there are still no trials to show the cardiovascular benefit of fibrates except for meta-analysis of high triglyceride and low HDL-C groups [36], and recently the Pemafibrate to Reduce Cardiovascular Outcomes by Reducing Triglycerides in Patients with Diabetes study has been discontinued as pemafibrate was reported to be unlikely to reduce CVD risk [37]. Additionally, niacin is recommended for treatment of hypertriglyceridemia in some guidelines, yet this agent is not recommended in combination with statin therapy due to a lack of additional benefits for CVD prevention in patients who have achieved LDL-C goals [29,30]. Pure eicosapentaenoic acid (EPA) can lower CVD risk in persons with moderately high triglyceride levels on statin therapy, which is the conclusion of the Japan EPA Lipid Intervention Study and the Reduction of Cardiovascular Events with Icosapent Ethyl–Intervention Trial [38,39]. Icosapent Ethyl is recommended to be used in combination with statins for specific patients with moderately high triglyceride levels in some guidelines [23,34]. Many agents for dyslipidemia treatment are under study. For example, acyl-coenzyme A: cholesterol acetyltransferase (ACAT) inhibitor was reported to decrease cholesteryl ester accumulation in macrophages in animal studies [40]. However, randomized controlled trials (RCT) using non-selective ACAT inhibitors failed to show benefits in the changes in coronary atheroma volume and carotid intima-media thickness [40], and no evidence from RCT for CVD outcomes is available.

However, some differences are also observed between guidelines issued by different countries and organizations at various time points. First, risk assessments are based on different lists of risk factors in different guidelines, even though some of the risk factors have neither enough data for CVD risk prediction and have not been evaluated in most risk-predictive models, nor an available interventional approach. As reported in our previous review on cardiovascular risk assessment [41], among the 10 guidelines on dyslipidemia, the risk-factor list ranged from five risk factors (including age, sex, systolic blood pressure, TC, and smoking) to 13 risk factors. Second, these guidelines recommend different algorithms to categorize CVD risk, which might be based on considerations of feasibility and practicability in local practice. An exponential model including 186 countries evaluated that using the same CVD risk estimation to initiate statins use, irrespective of age, sex, and country, is not appropriate globally. Considering current characteristics of the national population and safety in medication treatment to determine treatment strategy might be the optimal solution [42]. Third, different guidelines recommended different definitions of ‘high risk’. The varying recommendations for risk assessment among country-specific guidelines are generally central to treatment decision-making in clinical practice. However, these notable differences in the definition of high risk between guidelines may lead to an individual categorizing in a completely different risk bracket according to different guidelines developed by different organizations in different countries or regions, or even different guidelines developed in the same country or region. Fourth, the LDL-C treatment goals are also different among guidelines recommended by a comprehensive cardiovascular risk reduction strategy. In the 2019 European Society of Cardiology (ESC) / European Atherosclerosis Society (EAS) guidelines for the management of dyslipidemias [34], the LDL-C target goal is <55 mg/dL for individuals with very high risk in primary or secondary prevention. In patients with ASCVD who experienced a second vascular event within 2 years, it is recommended to lower LDL-C to less than 40 mg/dL (Table S1), which is consistent with the recommendations for patients who have extreme risk of Polish Lipid Association published in 2021 [27]. This further risk stratification for patients with ASCVD is considered in more and more countries. 

The initial doses of statins recommended by the guidelines also differ. Most country-specific guidelines recommend the dose of statins should be based on baseline ASCVD risk and expected LDL-C reduction of that risk [22,24,27,28,35], while some countries only mentioned the initial dose in very high and high risk individuals [25,26]. High-intensity statins or a maximally tolerated dose of statins are the most common therapeutic dose for individuals with very high and/or high CVD risk [20,21,25,30,32,33,34]. Moderate-intensity statins are recommended in Chinese guideline considering the safety of the high-intensity statins in the Chinese population [19]. 

Another aspect that merits special concern is the screening for genetic dyslipidemias. For example, as an important type of genetic dyslipidemias, screening for familial hypercholesterolemia (FH) has been recommended by the guidelines issued by the American Association of Clinical Endocrinologists and American College of Endocrinology, the National Institute for Health and Care Excellence, Japan Atherosclerosis Society, the Chinese Society of Cardiology and Philippine Heart Association [19,20,23,24,25,28,29,30,33,34,35]. However, the majority of national guidelines worldwide have no such recommendation, leaving room for enhanced detection of FH.

Inconsistencies in the recommendations for the management of dyslipidemia might be a contributing factor to low implementation in clinical practice. Additionally, for some countries in most parts of Africa and Eastern Europe, no local clinical practice guidelines exist for dyslipidemia.

### 4.2. Treatment and Control of Dyslipidemia

For the global population, advocating a healthy lifestyle, understanding the harm of dyslipidemia, and preventing the occurrence of dyslipidemia in early stages are the cornerstones of ASCVD prevention. For individuals with a high risk of ASCVD and patients with established ASCVD, the key point is to ensure the use of lipid-lowering drugs and improve the treatment and control rate of dyslipidemia. However, the treatment and control rates show great differences across various countries.

Substantial decreases in non-HDL-C levels and subsequent reductions in the ASCVD burden in high-income Western countries during the past 30 years were partly owing to the contribution of lipid-lowering therapy. One study used data from the National Health and Nutrition Examination Survey from 2011 to 2012 and found that the treatment and control rate of dyslipidemia in the United States of America (USA) reached 54.1% and 66.0%, respectively [43]. The use of lipid-lowering drugs, especially statins has increased, which may be attributable to the availability of generic statins and reduced drug prices in health care systems. A retrospective longitudinal cohort study conducted from 2002 to 2013 in 157,000 adults aged 40 years and older in the USA [44] demonstrated that statins use in the general population increased 79.8%, especially the proportion of generic statins, from 8.4% in 2002–2003 to 81.8% in 2012–2013. However, the uptake of statins was suboptimal among patients with established ASCVD, at 49.8% in 2002–2003 and 58.1% in 2012–2013. The total expenditures and out-of-pocket expenditures associated with statins decreased, and further substitution of brand-name statins to generic statins may yield greater savings. In addition to the cornerstone role of statins in reducing ASCVD risk, the role of lipid lowering treatment beyond statins cannot be ignored. There is considerable evidence supporting the benefits of nonstatin cholesterol-lowering medications in combination with statins, especially cholesterol absorption inhibitors (ezetimibe) and PCSK9 inhibitors [45,46]. Nonstatin use in the USA adult population has also increased by 124%, from 2.5% in 2002–2003 to 5.6% in 2012–2013, and 15.9% of high-intensity statin users also used nonstatin in 2012–2013 [47].

By contrast, among countries in East and Southeast Asia (for example, China and Malaysia) that have had substantial increases in non-HDL-C levels and dyslipidemia-related ASCVD risk over the past 30 years, the treatment and control rates are unsatisfied. In the China Chronic Disease and Risk Factor Surveillance conducted among 163,641 Chinese adults aged > 18 years from 2013 to 2014, 11.2% were at high or very high risk of ASCVD. Among them, 74.5% individuals with high risk and 93.2% individuals with very high risk did not achieve their LDL-C lowering targets. Among the population with high and very high ASCVD risk that did not achieve their LDL-C lowering targets, only 5.5% and 14.5% received lipid-lowering drugs, respectively [48]. Although several studies have shown that the rate of statin utilization in Chinese patients with acute myocardial infarction during hospital admission has improved substantially in recent years [49], low adherence of statin use was found after discharge. A nationwide registry study with 192 participating hospitals from 2014 to 2018 among 6523 Chinese patients with acute coronary syndrome and a history of myocardial infarction or revascularization found that 50.8% were receiving lipid-lowering therapy before hospitalization (statin monotherapy in 98.4%, combination therapy in 1.2%), and only 30.1% of patients had LDL-C < 70 mg/dL at admission [50]. These studies suggest that statins use is inadequate in these regions. This seems to be related to many factors, including the availability and affordability of medications in hospitals or clinics at different levels, quality of care from medical service providers, and adherence to treatment by patients. The availability of medications is of utmost importance. One nationwide study assessed the availability of lipid-lowering medications in a survey of 3041 primary care institutions from 2016 to 2017, which included 145 community health centers, 384 community health stations from urban areas, 243 township health centers, and 2269 village clinics from rural areas in 31 Chinese provinces [51]. The availability of statins at these primary care institutions was only 49.7%, and village clinics had the lowest statin availability (43.7%) among the four types of institutions. This study was the first to address this important issue in a nationwide survey in these regions. The marked deficiencies in statins availability at primary care institutions are not consistent with the health needs of the population and have implications for patients’ health, which may mostly restrict the impact of lipid-lowering medication on reducing the CVD burden. Except concerning the marked underuse of lipid-lowering drugs for those meeting treatment criteria, more importantly, lipid-lowering therapy would certainly result in a higher economic burden from a public health perspective. In Western countries, statins therapy is cost-effective or cost-saving, especially in people with high CVD risk [52,53,54]. In China, with the intervention of government policy in recent years, the cost-effectiveness of lipid-lowering medication has significantly improved in Chinese population [55,56]. 

Effective community-based prevention strategies that promote lifestyle modification (e.g., dietary improvement and regular physical activity) are also needed to control dyslipidemia in the whole population and prevent the occurrence of dyslipidemia at an early stage. Understanding dyslipidemia related CVD risk and regular monitoring of blood lipids is also crucial. A large survey from 2007 to 2010 conducted in 43,368 Chinese adults aged ≥ 18 years reported that the awareness rate for dyslipidemia was 31.01% [57]. These data suggest the need to raise awareness of dyslipidemia among the general population and clinicians and increase the capacity of primary care institutions to screen and diagnose dyslipidemia in community residents.

## 5. Summary

This review provides a comprehensive overview of the global dyslipidemia epidemic and its management. The current features of epidemics of dyslipidemia include: (1) Differences in age-standardized non-HDL-C levels have been diminishing across countries over the past four decades, yet marked geographic differences in non-HDL-C levels still exist. (2) The global trend is that distribution of high non-optimal cholesterol has changed from high-income countries to some developing countries in East Asia and Southeast Asia. (3) Different countries and regions are in different stages of transition with respect to ASCVD burden, with decreased IHD and ischemic stroke deaths attributable to high LDL-C levels in high-income Western countries and a greatly increased burden of IHD and ischemic stroke deaths across Asian countries during this period. (4) This overview of cardiovascular risk assessment and dyslipidemia management from a global perspective can potentially guide countries in the development of their own risk assessment models and formulation of recommendations in their own guidelines according to the local requirements. (5) Notable differences exist in the treatment and control rate of dyslipidemia between different regions and countries, which emphasizes the need for consistent efforts to increase the compliance with medical treatment.

## Data Availability

Not applicable.

## Supplementary Materials

The following supporting information can be downloaded at: https://www.mdpi.com/article/10.3390/jcm11216377/s1, Table S1: Differences of guideline recommendations for management of plasma lipid disorders [11,19,20,21,22,23,24,25,26,27,28,29,30,31,32,33,34,35,58].
