# Supplementary material for: Global Trends in the Epidemiology and Management of Dyslipidemia
Source: J Clin Med. 2022 Oct 28;11(21):6377. doi: 10.3390/jcm11216377 (PMC9656679; doi:10.3390/jcm11216377)
Supplement: Supplementary file 1 [file jcm-11-06377-s001.zip › jcm-1935904-supplementary.pdf]

Journal of Clinical Medicine

Review

Global Trends in Epidemiology and Management of Dyslipidemia

Tianxiao Liu, Dong Zhao * and Yue Qi *

Center for Clinical and Epidemiological Research, Beijing Anzhen Hospital, Capital Medical University, Beijing Institute of Heart, Lung and Blood Vessel Diseases, The Key Laboratory of Remodeling-Related Cardiovascular Diseases, Ministry of Education, Beijing Municipal Key Laboratory of Clinical Epidemiology, Beijing, China
* Correspondence: deezhao@vip.sina.com (D.Z.); qiyue_bjcn@mail.ccmu.edu.cn (Y.Q.)

Table S1. Differences of guideline recommendations for management of plasma lipid disorders.

Super-region/Guidelines	Definitions of Extreme Risk	Definitions of Very High Risk	Definitions of High Risk	Treatment Goals of LDL-C
**East and Southeast Asia**				
Chinese Society of Cardiology (2016) [1]	NA	ASCVD patients	1. LDL-C ≥ 4.9 mmol/L or TC ≥ 7.2 mmol/L<br>2. Diabetes patients [LDL-C: 1.8–4.9 mmol/L (or TC: 3.1–7.2 mmol/L) and age ≥ 40 years]<br>3. 10-year risk of ASCVD ≥ 10%	Very high risk: 70 mg/dL<br>High risk: 100 mg/dL
Philippine Heart Association (2020) [2]	NA	NA	NA	Individuals with clinical ASCVD: 55 mg/dL<br>FH without ASCVD or without major risk factor/target organ damage: 70 mg/dL<br>Individuals with DM: 100 mg/dL
Ministry of Health (Malaysia) (2017) [3]	NA	1. Established CVD<br>2. DM with proteinuria or with a major risk factor<br>3. CKD (Stage 4/5)	1. CKD (Stage 3)<br>2. DM without target organ damage<br>3. Very high levels of individual risk factors<br>4. 10-year risk for CVD > 20%	Very high risk: 70 mg/dL<br>High risk: 100 mg/dL
**South Asia**				
Lipid Association of India (2016) [4]	NA	1. Preexisting ASCVD<br>2. DM with evidence of target organ damage or ≥2 other major ASCVD risk factors<br>3. HoFH	10-year risk for ASCVD death, MI or stroke >15%	Very high risk: 50 mg/dL<br>High risk: 70 mg/dL
**High-income Asia Pacific**				

Japan Atherosclerosis Society (2017) [5]	NA	NA	10-year risk of CAD ≥ 9%	CAD patients who are also suffering from high risk conditions: 70 mg/dL<br>History of CAD: 100 mg/dL<br>High risk: 120 mg/dL
Korean Society of Lipid and Atherosclerosis (2018) [6]	NA	ASCVD patients	1. Carotid artery disease<br>2. Abdominal aneurysm<br>3. DM	Very high risk: 70 mg/dL<br>High risk: 100 mg/dL
Ministry of Health (Singapore) (2016) [7]	NA	1. Established CAD, atherosclerotic cerebrovascular disease, aortic aneurysm or peripheral artery disease<br>2. DM with CKD<br>3. FH	1. Individuals with moderate to severe CKD<br>2. Individuals with DM<br>3. 10-year risk of CAD > 20%	Very high risk: 80 mg/dL<br>High risk: 100 mg/dL
**Central Asia, Middle East and North Africa**				
Ministry of Public Health Qatar (2020) [8]	NA	NA	NA	Patients on high-intensity statin therapy: 70 mg/dL<br>Patients on moderate-intensity statin therapy: 100 mg/dL
**Central and Eastern Europe**				
Polish Lipid Association (2021) [9]	1. Pol-SCORE risk > 20%;<br>2. Serious ACS event *;<br>3. Status post-ACS and FH;<br>4. Status post-ACS in a patient with DM with additional risk factors	1. Pol-SCORE risk ≥ 10% and ≤20% in primary prevention;<br>2. Documented CVD	Pol-SCORE risk ≥ 5% and <10%	Extreme risk: 40 mg/dL<br>Very high risk: 55 mg/dL<br>High risk: 70 mg/dL
**High-income Western countries**				
AHA/ACC (2018) [10]	NA	Patients with clinical ASCVD who have a history of multiple major ASCVD events or 1 major ASCVD event and multiple high risk conditions	10-year risk of ASCVD > 20%	Very high-risk: 70 mg/dL
ACC Consensus (2022) [11]	NA	Patients with clinical ASCVD who have a history of multiple major ASCVD events or 1 major ASCVD event and multiple high risk conditions	NA	Very high risk: 55 mg/dL

AACE/ACE (2017) [12]	1. Progressive ASCVD including unstable angina in patients after achieving an LDL-C < 70 mg/dL<br>2. Established clinical CVD in patients with DM, CKD 3/4, or HeFH<br>3. History of premature ASCVD	1. Established or recent hospitalization for ACS, coronary, carotid or peripheral vascular disease, 10-year risk > 20%<br>2. DM or CKD 3/4 with 1 or more risk factor(s)<br>3. HeFH	1. ≥2 risk factors and 10-year risk 10–20%<br>2. DM or CKD stage 3/4 with no other risk factors	Extreme risk: 55 mg/dL<br>Very high risk: 70 mg/dL<br>High risk: 100 mg/dL
Canadian Cardiovascular Society (2021) [13]	NA	NA	Framingham risk score ≥ 20%	Secondary ASCVD prevention patients: 70 mg/dL
National Vascular Disease Prevention Alliance (Australia) (2012) [14]	NA	NA	5-year risk of CVD > 15%	Do not recommend establishing lipid-lowering target values
New Zealand Ministry of Health (2018) [15]	NA	NA	5-year risk of CVD ≥ 15%	Individuals with a five-year CVD risk ≥ 15%: 70 mg/dL
National Institute for Health and Care Excellence (2014) [16]	NA	NA	10-year risk of CVD ≥ 20%	Do not recommend establishing lipid-lowering target values
ESC/EAS (2019) [17]	NA	1. ASCVD patients<br>2. SCORE ≥ 10%<br>3. FH with ASCVD or with another major risk factor<br>4. Severe CKD<br>5. DM and target organ damage: ≥ 3 major risk factors; or early onset of T1DM of long duration (>20 years)	1. SCORE ≥ 5% and <10%<br>2. Markedly elevated single risk factors<br>3. FH without other major risk factors<br>4. Moderate CKD<br>5. DM without target organ damage, with DM duration ≥10 years or other additional risk factor	Patients with ASCVD who experience a second vascular event within 2 years: 40 mg/dL<br>Very high risk: 55 mg/dL<br>High risk: 70 mg/dL
**Sub-Saharan Africa**				
South African Heart Association (2018) [18]	NA	Framingham CVD risk > 30%	Framingham CVD risk > 15% and ≤30%	Very high risk: 70 mg/dL<br>High risk: 97mg/dL

Abbreviations: AACE, American Association of Clinical Endocrinologists; ACC, American College of Cardiology; ACE, American College of Endocrinology; ACS, acute coronary syndrome; AHA, American Heart Association; ASCVD, atherosclerotic cardiovascular disease; CAD, coronary artery disease; CKD, chronic kidney disease; CVD, cardiovascular disease; DM, diabetes mellitus; ESC, European Society of Cardiology; EAS, European Atherosclerosis Society; FH, familial hypercholesterolemia; HeFH, heterozygous familial hypercholesterolemia; HoFH, homozygous familial hypercholesterolemia; LDL-C, low-density lipoprotein cholesterol; MI, myocardial infarction; NA, not available; Pol-SCORE, SCORE tables calibrated for the Polish population; SCORE, Systematic Coronary Risk Estimation; T1DM, type 1 diabetes mellitus; TC, total cholesterol. * Serious ACS events: status post-ACS with another vascular incident within the past 2 years; status post-ACS and multilevel atherosclerosis; status post-ACS and multivessel coronary artery disease. Super-regions, regions were defined according to NCD-RisC report [19].

Reference

1. Joint Committee for Guideline, Revision. 2016 Chinese guidelines for the management of dyslipidemia in adults. *J. Geriatr. Cardiol.* **2018**, *15*, 1–29. https://doi.org/10.11909/j.issn.1671-5411.2018.01.011.

2. Gonzalez-Santos, L.E.; Oliva, R.; Jimeno, C.; Gonzales, E.; Margarita Balabagno, M.; Ona, D.; Cinco, J.E.; Baston, A.; Caole-Ang, I.; Fojas, M.; et al. Executive Summary of the 2020 Clinical Practice Guidelines for the Management of Dyslipidemia in the Philippines. *J. ASEAN Fed. Endocr. Soc.* **2021**, *36*, 5–11. https://doi.org/10.15605/jafes.036.01.01.
3. Malaysia Ministry of Health. Management of Dyslipidaemia 2017 5th Edition. Available online: http://www.acadmed.org.my/index.cfm?&menuid=67 (accessed on 1 July 2022).
4. Iyengar, S.S.; Puri, R.; Narasingan, S.N.; Wangnoo, S.K.; Mohan, V.; Mohan, J.C.; Misra, A.; Sriram, U.; Dalal, J.J.; Gupta, R.; et al. Lipid Association of India Expert Consensus Statement on Management of Dyslipidemia in Indians 2016: Part 1. *J. Assoc. Physicians India* **2016**, *64*, 7–52.
5. Kinoshita, M.; Yokote, K.; Arai, H.; Iida, M.; Ishigaki, Y.; Ishibashi, S.; Umemoto, S.; Egusa, G.; Ohmura, H.; Okamura, T.; et al. Japan Atherosclerosis Society (JAS) Guidelines for Prevention of Atherosclerotic Cardiovascular Diseases 2017. *J. Atheroscler. Thromb.* **2018**, *25*, 846–984. https://doi.org/10.5551/jat.GL2017.
6. Rhee, E.J.; Kim, H.C.; Kim, J.H.; Lee, E.Y.; Kim, B.J.; Kim, E.M.; Song, Y.; Lim, J.H.; Kim, H.J.; Choi, S.; et al. 2018 Guidelines for the Management of Dyslipidemia in Korea. *J. Lipid Atheroscler.* **2019**, *8*, 78–131. https://doi.org/10.12997/jla.2019.8.2.78.
7. Singapore Ministry of Health. MOH Clinical Practice Guidelines on Lipids. Available online: https://www.moh.gov.sg/hpp/doctors/guidelines/GuidelineDetails/cpgmed_lipids (accessed on 1 July 2022).
8. Ministry of Public Health Qatar. National Clinical Guideline: The Assessment and Management of Dyslipidaemia. Available online: https://www.moph.gov.qa/english/OurServices/eservices/Pages/Clinical-Guidelines.aspx (accessed on 1 July 2022).
9. Banach, M.; Burchardt, P.; Chlebus, K.; Dobrowolski, P.; Dudek, D.; Dyrbus, K.; Gasior, M.; Jankowski, P.; Jozwiak, J.; Klosiewicz-Latoszek, L.; et al. PoLA/CFPiP/PCS/PSLD/PSD/PSH guidelines on diagnosis and therapy of lipid disorders in Poland 2021. *Arch. Med. Sci.* **2021**, *17*, 1447–1547. https://doi.org/10.5114/aoms/141941.
10. Grundy, S.M.; Stone, N.J.; Bailey, A.L.; Beam, C.; Birtcher, K.K.; Blumenthal, R.S.; Braun, L.T.; de Ferranti, S.; Faiella-Tommasino, J.; Forman, D.E.; et al. 2018 AHA/ACC/AACVPR/AAPA/ABC/ACPM/ADA/AGS/APhA/ASPC/NLA/PCNA Guideline on the Management of Blood Cholesterol: A Report of the American College of Cardiology/American Heart Association Task Force on Clinical Practice Guidelines. *J. Am. Coll. Cardiol.* **2019**, *73*, e285–e350. https://doi.org/10.1016/j.jacc.2018.11.003.
11. Writing Committee; Lloyd-Jones, D.M.; Morris, P.B.; Ballantyne, C.M.; Birtcher, K.K.; Covington, A.M.; DePalma, S.M.; Minissian, M.B.; Orringer, C.E.; Smith, S.C., Jr.; et al. 2022 ACC Expert Consensus Decision Pathway on the Role of Nonstatin Therapies for LDL-Cholesterol Lowering in the Management of Atherosclerotic Cardiovascular Disease Risk: A Report of the American College of Cardiology Solution Set Oversight Committee. *J. Am. Coll. Cardiol.* **2022**, *80*, 1366–1418. https://doi.org/10.1016/j.jacc.2022.07.006.
12. Jellinger, P.S.; Handelsman, Y.; Rosenblit, P.D.; Bloomgarden, Z.T.; Fonseca, V.A.; Garber, A.J.; Grunberger, G.; Guerin, C.K.; Bell, D.S.H.; Mechanick, J.I.; et al. American Association of Clinical Endocrinologists and American College of Endocrinology Guidelines for Management of Dyslipidemia and Prevention of Cardiovascular Disease-Executive Summarycomplete Appendix to Guidelines. *Endocr. Pract.* **2017**, *23*, 479–497. https://doi.org/10.4158/EP171764.GL.
13. Pearson, G.J.; Thanassoulis, G.; Anderson, T.J.; Barry, A.R.; Couture, P.; Dayan, N.; Francis, G.A.; Genest, J.; Gregoire, J.; Grover, S.A.; et al. 2021 Canadian Cardiovascular Society Guidelines for the Management of Dyslipidemia for the Prevention of Cardiovascular Disease in Adults. *Can. J. Cardiol.* **2021**, *37*, 1129–1150. https://doi.org/10.1016/j.cjca.2021.03.016.
14. National Vascular Disease Prevention Alliance. Guidelines for primary cardiovascular disease prevention. Available online: https://www.heartfoundation.org.au/Conditions/Fp-Lipid-management (accessed on 1 July 2022).
15. Ministry of Health New Zealand. Cardiovascular Disease Risk Assessment and Management for Primary Care. Available online: https://www.health.govt.nz/system/files/documents/publications/cardiovascular-disease-risk-assessment-management-primary-care-feb18-v4_0.pdf (accessed on 1 July 2022).
16. National Institute for Health and Care Excellence. Cardiovascular disease: Risk assessment and reduction, including lipid modification. Available online: https://www.nice.org.uk/guidance/cg181 (accessed on 1 July 2022).
17. Mach, F.; Baigent, C.; Catapano, A.L.; Koskinas, K.C.; Casula, M.; Badimon, L.; Chapman, M.J.; De Backer, G.G.; Delgado, V.; Ference, B.A.; et al. 2019 ESC/EAS Guidelines for the management of dyslipidaemias: Lipid modification to reduce cardiovascular risk. *Eur. Heart J.* **2020**, *41*, 111–188. https://doi.org/10.1093/eurheartj/ehz455.
18. Klug, E.; Raal, F.J.; Marais, A.D.; Smuts, C.M.; Schamroth, C.; Jankelow, D.; Blom, D.J.; Webb, D.A. South African dyslipidaemia guideline consensus statement: 2018 update A joint statement from the South African Heart Association (SA Heart) and the Lipid and Atherosclerosis Society of Southern Africa (LASSA). *S. Afr. Med. J.* **2018**, *108*, 973–1000.
19. N.C.D. Risk Factor Collaboration. Repositioning of the global epicentre of non-optimal cholesterol. *Nature* **2020**, *582*, 73–77. https://doi.org/10.1038/s41586-020-2338-1.
